# Dibromidobis(*N*,*N*,*N*′,*N*′-tetra­methyl­thio­urea-κ*S*)cadmium(II)

**DOI:** 10.1107/S1600536810028102

**Published:** 2010-07-17

**Authors:** Sidra Nawaz, Sana Sadaf, Mohammed Fettouhi, Atif Fazal, Saeed Ahmad

**Affiliations:** a Department of Chemistry, University of Engineering and Technology, Lahore 54890, Pakistan; b Department of Chemistry, King Fahd University of Petroleum and Minerals, Dhahran 31261, Saudi Arabia

## Abstract

In the title compound, [CdBr_2_(C_5_H_12_N_2_S)_2_], the Cd^II^ atom lies on a twofold rotation axis. It exhibits a distorted tetra­hedral coordination environment defined by two S atoms of two tetra­methyl­thio­urea (tmtu) ligands and two bromide ions. The crystal structure is consolidated by C—H⋯N and C—H⋯S hydrogen bonds.

## Related literature

For crystallographic and spectroscopic studies of thio­urea complexes, see: Al-Arfaj *et al.* (1998 ▶); Ali *et al.* (2009 ▶); Isab *et al.* (2009 ▶); Lobana *et al.* (2008 ▶); Marcos *et al.* (1998 ▶); Moloto *et al.* (2003 ▶). The structure of the title compound is isotypic with [Cd(tmtu)_2_I_2_] (Nawaz *et al.*, 2010*a* ▶) and [Hg(tmtu)_2_Cl_2_] (Nawaz *et al.*, 2010*b* ▶).
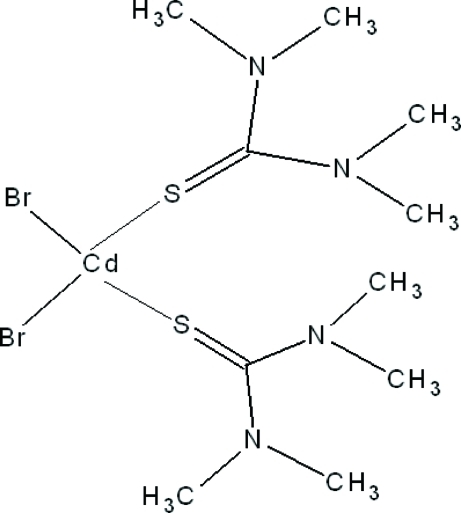


## Experimental

### 

#### Crystal data

[CdBr_2_(C_5_H_12_N_2_S)_2_]
*M* *_r_* = 536.67
Monoclinic, 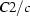

*a* = 18.6133 (17) Å
*b* = 10.0690 (9) Å
*c* = 13.4600 (12) Å
β = 130.834 (1)°
*V* = 1908.6 (3) Å^3^
*Z* = 4
Mo *K*α radiation
μ = 5.54 mm^−1^
*T* = 292 K
0.24 × 0.23 × 0.20 mm

#### Data collection

Bruker SMART APEX area-detector diffractometer
Absorption correction: multi-scan (*SADABS*; Sheldrick, 1996 ▶) *T*_min_ = 0.350, *T*_max_ = 0.404
12678 measured reflections
2379 independent reflections
2114 reflections with *I* > 2σ(*I*)
*R*_int_ = 0.028

#### Refinement

*R*[*F* ^2^ > 2σ(*F* ^2^)] = 0.021
*wR*(*F* ^2^) = 0.052
*S* = 1.05
2379 reflections
92 parameters
H-atom parameters constrained
Δρ_max_ = 0.44 e Å^−3^
Δρ_min_ = −0.45 e Å^−3^

### 

Data collection: *SMART* (Bruker, 2008 ▶); cell refinement: *SAINT* (Bruker, 2008 ▶); data reduction: *SAINT*; program(s) used to solve structure: *SHELXS97* (Sheldrick, 2008 ▶); program(s) used to refine structure: *SHELXL97* (Sheldrick, 2008 ▶); molecular graphics: *SHELXTL* (Sheldrick, 2008 ▶); software used to prepare material for publication: *SHELXTL*.

## Supplementary Material

Crystal structure: contains datablocks I, global. DOI: 10.1107/S1600536810028102/wm2371sup1.cif

Structure factors: contains datablocks I. DOI: 10.1107/S1600536810028102/wm2371Isup2.hkl

Additional supplementary materials:  crystallographic information; 3D view; checkCIF report

## Figures and Tables

**Table 1:** Selected bond lengths (Å)

Cd1—S1	2.5580 (6)
Cd1—Br1	2.5735 (3)

**Table 2:** Hydrogen-bond geometry (Å, °)

*D*—H⋯*A*	*D*—H	H⋯*A*	*D*⋯*A*	*D*—H⋯*A*
C2—H2*A*⋯N2	0.96	2.53	2.855 (4)	100
C5—H5*A*⋯S1	0.96	2.65	3.026 (3)	104

**Fig. 1.**
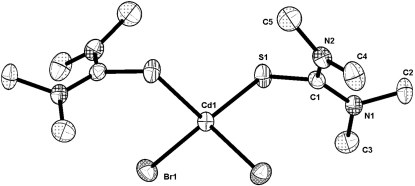
The molecular structure of title compound with the atomic numbering scheme. Displacement ellipsoids are drawn at the 30% probability level. H-atoms are omitted for clarity.

**Fig. 2.**
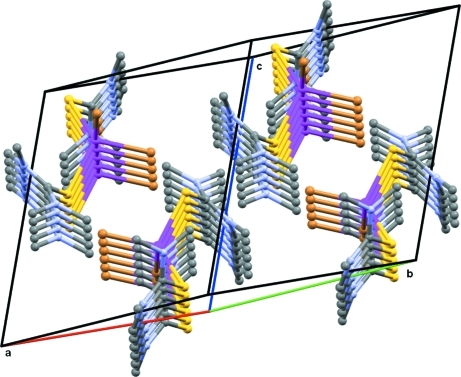
Packing diagram of the title complex

## supplementary crystallographic information

### Comment

The coordination chemistry of thioureas with metal ions has been the subject of several recent investigations because of their variable binding modes and because of the relevance of their binding sites to those in living systems. Crystallographic reports about *d*^10^ metal complexes of thioureas established that these ligands are coordinated *via* the sulfur atom (Al-Arfaj *et al.*, 1998; Moloto *et al.*, 2003). Spectroscopic data is also consistent with this finding (Isab *et al.*, 2009; Ali *et al.*, 2009). Herein, we report the crystal structure of a cadmium bromide complex with tetramethylthiourea (tmtu), [Cd(C_5_H_12_N_2_S_2_)_2_Br_2_], (I).

The crystal structure of (I) consists of discrete molecular species in which the cadmium atom is located on a twofold rotation axis (Fig. 1). It exhibits a distorted tetrahedral coordination environment defined by two tetramethylthiourea (tmtu) ligands and two bromide ions. The tmtu ligand is terminally bound to the Cd^II^ atom *via* coordination of the S1 atom. The Cd—S and Cd—Br bond lengths are 2.5580 (6) and 2.5735 (3) Å, respectively. These values are in agreement with those reported for related compounds, e.g. (Al-Arfaj *et al.*, 1998; Lobana *et al.* 2008; Marcos *et al.*, 1998; Moloto *et al.*, 2003). The bond angles around Cd are indicative of a slight tetrahedral distortion, with the S—Cd—S angle showing the largest deviation (117.70 (3)°) from the ideal value. The SCN_2_— moiety of the tmtu ligand is essentially planar, the maximum deviation from the mean plane being 0.007 (2) Å for the carbon atom. The fragments N1—C1—C2—C3 and N2—C1—C4—C5 are also close to planarity. The maximum deviations from the mean planes are 0.072 (2) Å and 0.065 (2) Å for N1 and N2, respectively. These values are consistent with a significant C—N double bond character and electron delocalization in the SCN_2_— moiety. The steric effect of the two adjacent 1,3-methyl groups imposes a dihedral angle of 47.4 (2) ° for the two mean planes and therefore a tilted conformation. The latter is likely stabilized by non-classical intramolecular hydrogen bonding interactions involving methyl H atoms with sulfur and nitrogen atoms (C2—H2A ···N2 and C5—H5A···S1). The molecules pack to form columns approximately parallel to [110] direction (Fig. 2).

The structure of (I) is isotypic with [Cd(tmtu)_2_I_2_] (Nawaz *et al.*, 2010*a*), with an equivalent degree of distortion from the ideal tetrahedral configuration and similar Cd—S and C—N bond lengths. The tmtu bond lengths are also consistent with those found for the likewise isotypic compound [Hg(tmtu)_2_Cl_2_] (Nawaz *et al.*, 2010*b*), in which a significantly higher distortion of the metal ion coordination sphere is observed.

### Experimental

0.35 g (1.0 mmol) cadmium(II) bromide tetrahydrate dissolved in 10 ml water were added to two equivalents of tetramethylthiourea in methanol. A white precipitate formed and was filtered off. The filtrate was kept for crystallization. As a result, an off-white crystalline product suitable for single crystal X-ray diffraction was obtained.

### Refinement

H atoms were placed in calculated positions with a C—H distance of 0.96 Å and *U*_iso_(H) = 1.5 *U*_eq_(C).

### Crystal data

**Table:**

[CdBr_2_(C_5_H_12_N_2_S)_2_]	*F*(000) = 1048
*M**_r_* = 536.67	*D*_x_ = 1.868 Mg m^−^^3^
Monoclinic, *C*2/*c*	Mo *K*α radiation, λ = 0.71073 Å
Hall symbol: -C 2yc	Cell parameters from 12678 reflections
*a* = 18.6133 (17) Å	θ = 2.5–28.3°
*b* = 10.0690 (9) Å	µ = 5.54 mm^−^^1^
*c* = 13.4600 (12) Å	*T* = 292 K
β = 130.834 (1)°	Block, colorless
*V* = 1908.6 (3) Å^3^	0.24 × 0.23 × 0.20 mm
*Z* = 4	

### Data collection

**Table:**

Bruker SMART APEX area-detector diffractometer	2379 independent reflections
Radiation source: normal-focus sealed tube	2114 reflections with *I* > 2σ(*I*)
graphite	*R*_int_ = 0.028
ω scans	θ_max_ = 28.3°, θ_min_ = 2.5°
Absorption correction: multi-scan (*SADABS*; Sheldrick, 1996)	*h* = −24→24
*T*_min_ = 0.350, *T*_max_ = 0.404	*k* = −13→13
12678 measured reflections	*l* = −17→17

### Refinement

**Table:**

Refinement on *F*^2^	Secondary atom site location: difference Fourier map
Least-squares matrix: full	Hydrogen site location: inferred from neighbouring sites
*R*[*F*^2^ > 2σ(*F*^2^)] = 0.021	H-atom parameters constrained
*wR*(*F*^2^) = 0.052	*w* = 1/[σ^2^(*F*_o_^2^) + (0.025*P*)^2^ + 1.3001*P*] where *P* = (*F*_o_^2^ + 2*F*_c_^2^)/3
*S* = 1.05	(Δ/σ)_max_ = 0.001
2379 reflections	Δρ_max_ = 0.44 e Å^−^^3^
92 parameters	Δρ_min_ = −0.45 e Å^−^^3^
0 restraints	Extinction correction: *SHELXTL* (Sheldrick, 2008), Fc^*^=kFc[1+0.001xFc^2^λ^3^/sin(2θ)]^-1/4^
Primary atom site location: structure-invariant direct methods	Extinction coefficient: 0.0068 (2)

### Special details

**Table:**

Geometry. All e.s.d.'s (except the e.s.d. in the dihedral angle between two l.s. planes) are estimated using the full covariance matrix. The cell e.s.d.'s are taken into account individually in the estimation of e.s.d.'s in distances, angles and torsion angles; correlations between e.s.d.'s in cell parameters are only used when they are defined by crystal symmetry. An approximate (isotropic) treatment of cell e.s.d.'s is used for estimating e.s.d.'s involving l.s. planes.
Refinement. Refinement of *F*^2^ against ALL reflections. The weighted *R*-factor *wR* and goodness of fit *S* are based on *F*^2^, conventional *R*-factors *R* are based on *F*, with *F* set to zero for negative *F*^2^. The threshold expression of *F*^2^ > σ(*F*^2^) is used only for calculating *R*-factors(gt) *etc*. and is not relevant to the choice of reflections for refinement. *R*-factors based on *F*^2^ are statistically about twice as large as those based on *F*, and *R*- factors based on ALL data will be even larger.

### Fractional atomic coordinates and isotropic or equivalent isotropic displacement parameters (Å^2^)

**Table:**

	*x*	*y*	*z*	*U*_iso_*/*U*_eq_	
Cd1	1.0000	0.70441 (2)	0.2500	0.03969 (8)	
Br1	1.149352 (17)	0.56647 (3)	0.34643 (3)	0.05549 (9)	
S1	1.03619 (4)	0.83583 (7)	0.44089 (5)	0.04794 (14)	
N1	0.91758 (14)	0.76405 (19)	0.4771 (2)	0.0474 (4)	
N2	0.85096 (13)	0.8900 (2)	0.29223 (18)	0.0471 (4)	
C1	0.92634 (14)	0.82923 (19)	0.39910 (19)	0.0366 (4)	
C2	0.8527 (2)	0.8077 (3)	0.4968 (3)	0.0672 (7)	
H2A	0.8267	0.8928	0.4562	0.101*	
H2B	0.8867	0.8145	0.5891	0.101*	
H2C	0.8022	0.7444	0.4580	0.101*	
C3	0.9861 (2)	0.6637 (3)	0.5706 (3)	0.0724 (8)	
H3A	1.0111	0.6189	0.5359	0.109*	
H3B	0.9552	0.6004	0.5851	0.109*	
H3C	1.0369	0.7056	0.6522	0.109*	
C4	0.75337 (18)	0.8418 (3)	0.2186 (3)	0.0694 (8)	
H4A	0.7550	0.7542	0.2480	0.104*	
H4B	0.7211	0.8394	0.1266	0.104*	
H4C	0.7203	0.9005	0.2330	0.104*	
C5	0.8609 (2)	0.9921 (3)	0.2254 (3)	0.0697 (8)	
H5A	0.9218	1.0340	0.2870	0.105*	
H5B	0.8118	1.0573	0.1892	0.105*	
H5C	0.8557	0.9524	0.1561	0.105*	

### Atomic displacement parameters (Å^2^)

**Table:**

	*U*^11^	*U*^22^	*U*^33^	*U*^12^	*U*^13^	*U*^23^
Cd1	0.04286 (13)	0.04283 (13)	0.04311 (13)	0.000	0.03237 (11)	0.000
Br1	0.05068 (15)	0.05784 (16)	0.05843 (16)	0.01407 (10)	0.03588 (13)	0.00638 (11)
S1	0.0391 (3)	0.0665 (4)	0.0425 (3)	−0.0082 (2)	0.0286 (2)	−0.0132 (2)
N1	0.0572 (11)	0.0471 (10)	0.0559 (11)	0.0041 (9)	0.0448 (10)	0.0036 (8)
N2	0.0448 (10)	0.0540 (11)	0.0427 (10)	0.0038 (8)	0.0287 (9)	−0.0020 (8)
C1	0.0406 (10)	0.0363 (10)	0.0371 (10)	−0.0014 (8)	0.0272 (9)	−0.0061 (8)
C2	0.0799 (19)	0.0800 (19)	0.0815 (19)	−0.0012 (15)	0.0702 (18)	−0.0047 (15)
C3	0.090 (2)	0.0600 (16)	0.082 (2)	0.0174 (15)	0.0627 (19)	0.0238 (15)
C4	0.0393 (13)	0.101 (2)	0.0573 (16)	0.0026 (14)	0.0268 (12)	−0.0123 (15)
C5	0.0804 (19)	0.0721 (18)	0.0571 (15)	0.0165 (15)	0.0451 (15)	0.0201 (14)

### Geometric parameters (Å, °)

**Table:**

Cd1—S1	2.5580 (6)	C2—H2B	0.9600
Cd1—S1^i^	2.5580 (6)	C2—H2C	0.9600
Cd1—Br1^i^	2.5735 (3)	C3—H3A	0.9600
Cd1—Br1	2.5735 (3)	C3—H3B	0.9600
S1—C1	1.731 (2)	C3—H3C	0.9600
N1—C1	1.335 (3)	C4—H4A	0.9600
N1—C3	1.460 (3)	C4—H4B	0.9600
N1—C2	1.463 (3)	C4—H4C	0.9600
N2—C1	1.331 (3)	C5—H5A	0.9600
N2—C5	1.455 (3)	C5—H5B	0.9600
N2—C4	1.471 (3)	C5—H5C	0.9600
C2—H2A	0.9600		
			
S1—Cd1—S1^i^	117.70 (3)	H2A—C2—H2C	109.5
S1—Cd1—Br1^i^	105.899 (14)	H2B—C2—H2C	109.5
S1^i^—Cd1—Br1^i^	106.524 (15)	N1—C3—H3A	109.5
S1—Cd1—Br1	106.524 (15)	N1—C3—H3B	109.5
S1^i^—Cd1—Br1	105.899 (14)	H3A—C3—H3B	109.5
Br1^i^—Cd1—Br1	114.676 (17)	N1—C3—H3C	109.5
C1—S1—Cd1	100.04 (7)	H3A—C3—H3C	109.5
C1—N1—C3	122.1 (2)	H3B—C3—H3C	109.5
C1—N1—C2	122.3 (2)	N2—C4—H4A	109.5
C3—N1—C2	114.2 (2)	N2—C4—H4B	109.5
C1—N2—C5	121.5 (2)	H4A—C4—H4B	109.5
C1—N2—C4	122.8 (2)	N2—C4—H4C	109.5
C5—N2—C4	114.6 (2)	H4A—C4—H4C	109.5
N2—C1—N1	119.41 (19)	H4B—C4—H4C	109.5
N2—C1—S1	121.32 (16)	N2—C5—H5A	109.5
N1—C1—S1	119.26 (16)	N2—C5—H5B	109.5
N1—C2—H2A	109.5	H5A—C5—H5B	109.5
N1—C2—H2B	109.5	N2—C5—H5C	109.5
H2A—C2—H2B	109.5	H5A—C5—H5C	109.5
N1—C2—H2C	109.5	H5B—C5—H5C	109.5

Symmetry codes: (i) −*x*+2, *y*, −*z*+1/2.

### Hydrogen-bond geometry (Å, °)

**Table:**

*D*—H···*A*	*D*—H	H···*A*	*D*···*A*	*D*—H···*A*
C2—H2A···N2	0.96	2.53	2.855 (4)	100
C5—H5A···S1	0.96	2.65	3.026 (3)	104
